# Serum Cytokine Profiles Associated with Specific Adjuvants Used in a DNA Prime-Protein Boost Vaccination Strategy

**DOI:** 10.1371/journal.pone.0074820

**Published:** 2013-09-03

**Authors:** Rachel Buglione-Corbett, Kimberly Pouliot, Robyn Marty-Roix, Kim West, Shixia Wang, Egil Lien, Shan Lu

**Affiliations:** Department of Medicine, University of Massachusetts Medical School, Worcester, Massachusetts, United States of America; University of Cape Town, South Africa

## Abstract

In recent years, heterologous prime-boost vaccines have been demonstrated to be an effective strategy for generating protective immunity, consisting of both humoral and cell-mediated immune responses against a variety of pathogens including HIV-1. Previous reports of preclinical and clinical studies have shown the enhanced immunogenicity of viral vector or DNA vaccination followed by heterologous protein boost, compared to using either prime or boost components alone. With such approaches, the selection of an adjuvant for inclusion in the protein boost component is expected to impact the immunogenicity and safety of a vaccine. In this study, we examined in a mouse model the serum cytokine and chemokine profiles for several candidate adjuvants: QS-21, Al(OH)_3_, monophosphoryl lipid A (MPLA) and ISCOMATRIX™ adjuvant, in the context of a previously tested pentavalent HIV-1 Env DNA prime-protein boost formulation, DP6-001. Our data revealed that the candidate adjuvants in the context of the DP6-001 formulation are characterized by unique serum cytokine and chemokine profiles. Such information will provide valuable guidance in the selection of an adjuvant for future AIDS vaccine development, with the ultimate goal of enhancing immunogenicity while minimizing reactogenicity associated with the use of an adjuvant. More significantly, results reported here will add to the knowledge on how to include an adjuvant in the context of a heterologous prime-protein boost vaccination strategy in general.

## Introduction

Recently, the RV144 clinical trial using a viral vector prime-recombinant protein boost vaccine demonstrated a low level, but statistically significant protection against HIV-1 infection in Thai volunteers [1–4]. The RV144 trial employed a canarypox viral vector ALVAC, encoding HIV-1 antigens env, gag, and pol, as a prime, followed by a boost with bi-clade AIDSVAX B/E recombinant gp120 protein boost formulated with adjuvant Al(OH)_3_. After extensive follow-up studies, new data are emerging to suggest that antibodies against certain critical areas of HIV-1 envelope proteins are responsible for protection; however, the antibody responses in the RV144 trial were not long lasting, which may have led to reduced protection during the clinical trial observation period.

Following the results of the RV144 trial, especially given the renewed interest of including recombinant Env proteins in the future HIV-1 vaccine development, the study of adjuvants is gaining more momentum as adjuvant is a critical component of most licensed recombinant protein-based human vaccine. A more immunogenic adjuvant than Al(OH)_3_ may provide enhanced and long lasting antibody responses, which may improve the level of protection compared to that observed in the RV144 trial.

A key consideration in vaccine development is enhancing immunogenicity while mitigating associated adverse effects. The inclusion of adjuvants in vaccine formulations has long been a method of improving vaccine efficacy, but these adjuvants are not without risk of eliciting local and systemic adverse effects. HIV-1 recombinant Env protein-based vaccines are now primarily being used as a boost component in emerging HIV-1 vaccine strategies. Thus, it is very important scientifically to examine how an adjuvant works when it is formulated with Env proteins, and in the context of hosts who have been primed by a gene-based vaccine (such as viral vector or DNA vaccine) encoding antigens similar to the boost.

Our group has demonstrated the high immunogenicity of the pentavalent DNA prime-protein boost HIV-1 vaccine formulation, DP6-001, in preclinical and phase I clinical trials [5–7]. The saponin adjuvant QS-21, derived from *Quillaia saponaria*, was included as part of the Env protein boost in the phase I clinical study of DP6-001 formulation. Previously published studies have also utilized QS-21 as part of recombinant Env protein alone vaccines in humans, demonstrating a potent immunogenicity allowing for reduced antigen dose, and characterized by improved binding and neutralizing antibody responses [8,9]. However, in these studies QS-21 has been associated with serious local reactions, including pain, induration, erythema, as well as systemic adverse effects such as hypertension, myalgia, headache, and vasovagal episodes which were rare and not proven to be caused by QS-21 directly [8,9]. We also observed in clinical trials of DP6-001, local skin reactions as well as rare skin based vasculitis associated with QS-21 adjuvanted protein boosting [6].

In the current study, the serum cytokine profiles in mice in the context of the DP6-001 DNA prime-protein boost were analyzed, in the context of several protein-adjuvant boost formulations. MPLA and ISCOMATRIX™ adjuvant were examined in formulation with the DP6-001 vaccine, along with QS-21 and aluminum hydroxide (Al(OH)_3_), which were tested in previous clinical studies of HIV-1 vaccines. Similar to QS-21, ISCOMATRIX™ adjuvant consists of a *Quillaia* saponin fraction, which is mixed with cholesterol and phospholipid under controlled conditions to form a cage-like structure of approximately 40nm in diameter. The ISCOMATRIX™ adjuvant can then be formulated with virtually any antigen to make a vaccine. Formulating saponin with cholesterol and phospholipid appears to ameliorate the reactogenicity associated with using free saponin, as ISCOMATRIX™ adjuvant retains the immunogenic potency and antigen dose reduction potential of saponin but demonstrates improved tolerability [10,11]. ISCOMATRIX™ adjuvant promotes a balanced Th1/Th2 response, as well as a uniquely strong cytotoxic T cell response and long-lasting antibody responses in both animal and human models [12–15]. This broad, robust activation of adaptive immunity has made ISCOMATRIX™ adjuvant particularly efficacious in clinical studies of anti-tumor vaccines [16–18], as well as trials of therapeutic and protective human papilloma virus (HPV) vaccines and influenza vaccines [19,20]. Comprehensive clinical safety data has been compiled and reported for six clinical trials of ISCOMATRIX™ adjuvant with a variety of antigen formulations [11].

MPLA is low-toxicity, dephosphorylated derivative of lipopolysaccharide (LPS) [21]. MPLA, like LPS, acts via Toll-like receptor (TLR) 4, but results in significantly less inflammation compared to its parent molecule [21,22]. As an adjuvant, MPLA improves vaccine-specific antibody responses, as well as induces potent Th1 responses in preclinical and clinical models, resulting in a complex inflammatory response consisting of neutrophils, antigen-presenting cells (APCs), and natural killer cells (NKs). Despite its potency, MPLA has been well tolerated in clinical trials. Currently, MPLA is a component of the licensed GlaxoSmithKline (GSK) human vaccines Fendrix® for hepatitis B in combination with aluminum phosphate and, in formulation with aluminum hydroxide in Adjuvant System AS04, in Cervarix® for human papilloma virus [21–23]. MPLA has also been clinically evaluated in GSK’s AS01 adjuvant system in combination with QS-21 as a component of a malaria vaccine [24]. Aluminum-based adjuvants are the most widely utilized in human vaccines due to their tolerability and consistent induction of humoral immunity. Thus, Al(OH)_3_ has been included in the current study to provide a baseline control in the generation of an acceptable profile of tolerability [25,26].

The relative immunogenicity of our candidate adjuvants in combination with HIV Env proteins was assessed in both C57Bl/6 and Balb/c mice, based on a comprehensive profile of vaccine-specific antibody and T cell responses, as well as non-antigen specific serum cytokines detected shortly after protein immunization. While results show that the candidate adjuvants demonstrated comparable vaccine-specific immunogenic potency by IgG ELISA and T cell ELISpot, the analysis of serum cytokines allowed us to distinguish a profile of characteristic cytokines for each adjuvant. With this information, we will be better informed in the future for the selection of an adjuvant as part of a prime-boost HIV-1 vaccine formulation such as the polyvalent DNA prime- Env protein boost shown in the current report.

## Materials and Methods

### Ethics Statement

This study was carried out in strict accordance with the recommendations in the Guide for the Care and Use of Laboratory Animals of the National Institutes of Health. The protocol was approved by the Institutional Animal Care and Use Committee of the University of Massachusetts Medical School (Protocol Number: A-925). Termination was performed under anesthesia, and all efforts were made to minimize suffering.

### HIV-1 gp120 DNA vaccine

The gp120-expressing DNA vaccine component of the DP6-001 formulation was composed of equal amounts of five plasmids encoding codon-optimized gp120 genes from primary HIV-1 isolates: A (92UG037.8), B (92US715.6), Ba-L, Czm (96ZM651), and E (93TH976.17) in the common vector pSW3891 as previously described [27]. DNA vaccine plasmids were grown up in HB101 strain of *E. coli*, and prepared using a Plasmid Giga Kit (Qiagen, Valencia, CA). Endotoxin-free DNA plasmids were prepared using EndoFree Plasmid Mega Kit (Qiagen). According to Qiagen technical specifications, EndoFree Plasmid Kits yield DNA plasmids associated with <0.1 EU/ μg DNA, while standard Plasmid Kits are estimated at 9.3 EU/μg DNA. DNA plasmid expression was confirmed by transient expression in 293T cells and Western blot.

### HIV-1 gp120 Protein/Adjuvant Formulations

The protein component of DP6-001 was composed of equal parts of five recombinant gp120 proteins homologous to DNA vaccine components. These gp120 proteins were produced in CHO cell lines by Advanced Bioscience Laboratory, Inc. (ABL) as previously described [7]. Final protein product consisted of 7 µg/ per gp120 protein at each immunization, in Dulbecco’s phosphate buffered saline (DPBS) (Gibco, Invitrogen, Grand Islands, NY). Protein mixes were formulated prior to immunization with the candidate adjuvants: 5 µg QS-21, 175 µg Al(OH)_3_ gel (Sigma Aldrich Corp., St. Louis, MO), 25 µg synthetic MPLA (Avanti Polar Lipids, Inc., Alabaster, AL), or 1.5 ISCO™ Units of ISCOMATRIX™ adjuvant (CSL Limited, Parkville, Victoria, Australia). 1.5 ISCO™ Units is equivalent to 1.5 µg of ISCOPREP™ saponin and to allow easier comparison with other adjuvants the µg measurement is used in this article.

### Animal Immunizations

**Figure 1:**
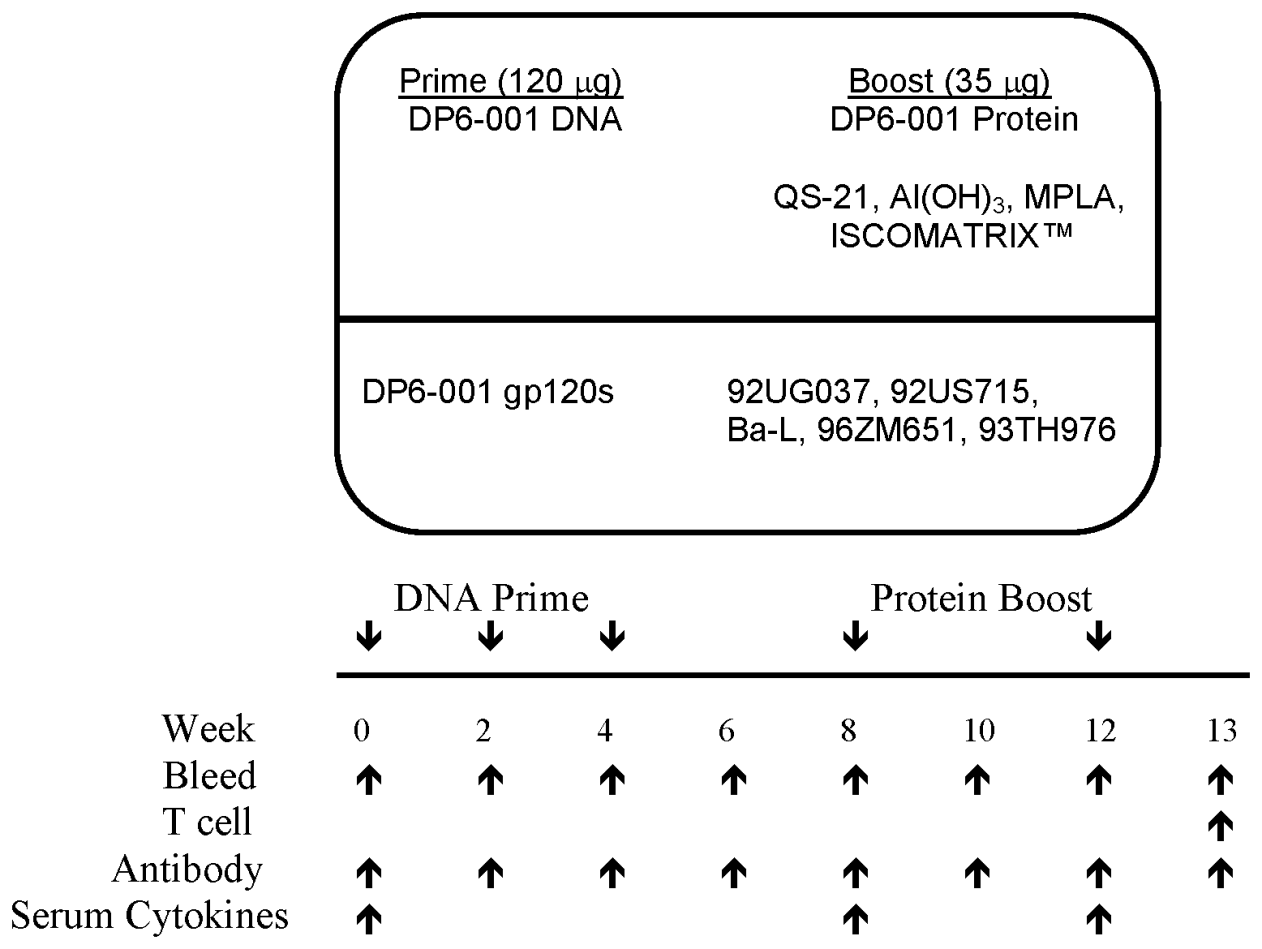
Study design and immunization schedule. C57Bl/6 mice were immunized with three pentavalent DNA primes followed by two heterologous gp120 protein boost. The pentavalent vaccine mixture of both DNA and protein components consisted of HIV-1 Env from clades A (92UG037.8), B (92US715.6 and Bal), C (96ZM651), and E (93TH976.17). DNA and protein doses indicated are for a total of five immunogens at each immunization. Four adjuvants were tested individually as part of the protein boost. Time points of immunizations, and sample collections for different assays were indicated.

Balb/c (6-8 weeks old, mixed sex) and C57Bl/6 (6-8 weeks old, mixed sex) mice were obtained from Taconic Farms and maintained in Department of Animal Medicine animal facility at University of Massachusetts Medical School, according to an IACUC-approved protocol. Mice received intramuscular (i.m.) DNA immunizations of total 120 µg gp120 DNA plasmid at each immunization, divided between each quadriceps at weeks 0, 2, and 4. Sera were collected following each immunization. After the third DNA immunization, sera were collected 6 hours following immunization. Mice received protein boosts of 7 µg/mouse per gp120 protein for a total protein immunization of 35 µg at each immunization. Protein boosts were formulated with candidate adjuvants as described above, and administered via two i.m. injections divided between each quadriceps at weeks 8 and 12. Mice were bled multiple times for serum antibody and cytokine analysis (Figure 1). Mice were terminated 7 days after the final protein boost according to an IACUC approved procedure, at which time sera and spleens were collected.

### Enzyme linked immunosorbent assay (ELISA)

gp120-specific antibody responses were assessed by ELISA, performed as previously described with some modifications [28]. Briefly, 96-well EIA/RIA microtiter plates (Costar, Corning, NY) were coated with 5 µg/well ConA diluted in PBS for 1 hr. Between each step, plates were washed with PBS and 0.1% Triton X-100 five times using AquaMax2000 automatic plate washer (Molecular Devices, Sunnyvale, CA). Plates were coated with 1 µg/ml of the five recombinant gp120 protein mix used in immunizations. Wells were blocked overnight (4% whey by weight whey dilution buffer and 5% powdered milk) at 4^o^C. Plates were incubated with 100 µl of serially diluted mouse sera in duplicate for 1 hr. Biotinylated anti-mouse IgG (Vector Laboratories, Burlingame, CA) was added at 1.5 µg/ml for 1 hr. Horseradish-peroxidase (HRP)-conjugated streptavidin (Vector Laboratories) at 0.5 µg/ml was added and incubated for 1 hr. Plates were developed with TMB substrate (Sigma-Aldrich, St. Louis, MO) for 5 minutes, followed by the addition of 2 N H_2_SO_4_. Optical density (OD) of 450 nm (OD_450_) minus the background of plate absorbance at 630 nm, was read on a Multiskan FC (Thermo Fischer Scientific, Waltham, MA). The endpoint titer was determined as the highest dilution at which the OD_450_ equaled twice the OD_450_ of negative control wells. Statistically significant differences between titers were analyzed using Student’s *t* test.

For temporal antibody responses, pooled mouse sera dilutions of 1:250 or 1:500 were prepared for each collection time point. For mouse IgG isotyping ELISA, standard curve wells were coated with 0.5 µg/ml IgG2c or IgG1 coating antibody (Southern Biotech, Birmingham, AL) at 1:3 serial dilutions from a starting dilution of 1:1000. Biotin-conjugated IgG2c or IgG1 detection antibody (Southern Biotech) was applied at 0.5 µg/ml. Plates were washed, developed, and endpoint titers were determined as described above.

### Splenocyte preparation

Spleens were harvested 7 days following the second protein boost. Spleens were homogenized in complete RPMI media, with 10% heat-inactivated FBS (HyClone, Logan, UT), and 1% Penicillin-Streptomycin. Single-cell suspensions were made by pressing each spleen through a screen, and washing with media. Red blood cells were lysed with Red Blood Cell Lysis Buffer (Sigma). Cells were washed, counted, and diluted to a final concentration of 1x10^7^ cells/ml.

### Intracellular cytokine staining

All fluorophore-conjugated antibodies, unless otherwise noted, were obtained from BD Pharmingen (San Diego, CA). Splenocytes were cultured in 96-well cell culture round-bottom plates (Costar) at 1x10^6^ cells per well. Splenocytes were co-incubated with 2 µg/ml human IL-2, GolgiPlug (BD Biosciences, San Diego, CA), and peptide. Positive controls were stimulated with BD Leukocyte Activating Cocktail (BD Pharmingen, San Diego, CA). Antigen-specific stimulation consisted peptides 8771-8886 from a consensus clade B peptide pool (Cat. No. 9480, NIH AIDS Research & Reference Program, Germantown, MD) covering the region of gp120, composed of 115 15mer peptides overlapping by 11 amino acids each, at an individual peptide concentration of 2 µg/ml. Mock-stimulated splenocytes were treated with media, hIL-2, and GolgiPlug alone. Splenocytes were incubated at 37^o^C for 5 hr, after which cells were washed in 2% FBS/PBS staining buffer. Non-specific binding was blocked by incubating with 5 µg/ml α-Fcγ R III/II (2.4 G2) antibody (BD Pharmingen) at 4^o^C for 10 minutes. Cells were washed in staining buffer, and then incubated with LIVE/DEAD Fixable Blue (Invitrogen, Carlsbad, CA). Cells were washed in staining buffer, and then incubated with anti-CD4-Alexa700 and anti-CD8-PerCPCy5.5 at 0.4 µg/ml for 20 minutes at 4^o^C. Cells were washed in staining buffer, and were then fixed and permeabilized in Cytofix/Cytoperm (BD Biosciences) in the dark at 4^o^C for 20 minutes. Cells were washed in 1X PermWash (BD Biosciences). Cells were stained with anti-IFNγ-FITC, anti-IL-2-PECy7, and anti-IL-6-PE, diluted in 1X Permwash, for 30 minutes at 4^o^C. Cells were washed in Permwash, and resuspended in staining buffer. Stained splenocytes were analyzed on an LSRII FACS machine (BD Biosciences, San Jose, CA), and data was analyzed using FlowJo software (Treestar, Ashland, OR).

### T Cell ELISpot

ELISpot reagents (IL-2, IL-4 IFNγ) were obtained from Mabtech (Mariemont, OH) or from BD Biosciences (IL-6) (San Diego, CA). ELISpots were performed according to manufacturer’s instructions. Pre-coated MSIP PVDF-plates (Millipore, Billerica, MA) were seeded with splenocytes from immunized mice (prepared as above) at a 2.5x10^5^ cells/well. Positive controls were stimulated with 20 ng/ml phorbol 12-myristate 13-acetate (PMA) (Sigma-Aldrich, St. Louis, MO) and 500 ng/ml ionomycin (Sigma-Aldrich). Antigen-specific stimulation was performed with truncated peptide pools derived from clade B consensus Env including the V2/V3 pool (8836-8844) [29] (provided by NIH AIDS Reagent Repository), at an individual peptide concentration of 2 µg/ml. Mock-stimulated wells received media only. Plates were incubated 18-20 hr at 37^o^C in 5% CO_2_. Positive spots were visualized on a CTL Imager and counting was performed with Immunospot^TM^ software (Cellular Technology Ltd., Shaker Heights, OH)

### Luminex

Cytokines and chemokines were quantified in serum collected from individual mice prior to immunization at week 0, and 6 hr following each protein boost at weeks 8 and 12, using a custom Bio-Plex cytokine assay (Bio-Rad, Hercules, CA) according to manufacturer’s instructions. The panel of cytokines and chemokines included: IFNγ, IL-1β, IL-2, IL-4, IL-6, Eotaxin, G-CSF, KC, MIP-1α, MIP-1β, MCP-1, and RANTES. After collection, serum samples were stored at -80^o^C until the conclusion of the study, and all serum samples from each time point of interest were run in a single Luminex experiment. Prior to assay, serum samples were diluted 1:4 in sample diluent. Samples were read on a Bio-Plex 200 system with Bio-Plex Manager software (Bio-Rad).

### Statistical analysis

All data is presented as the mean of individual mice +/- standard error of the mean (SEM). Statistical analysis was performed using a Student’s *t* test, a one-way ANOVA followed by a Tukey post-test, or a two-way ANOVA followed by a Bonferonni post-test. 

## Results

### Study Design and Immunization Schedule

Wild type C57Bl/6 and Balb/c mice were immunized with DP6-001 gp120 vaccines formulated with different adjuvants based on a dose and schedule from previously completed clinical studies (Figure 1). Mice were primed i.m. three times with pentavalent gp120 DNA plasmids at weeks 0, 2, and 4 and boosted with two matched pentavalent gp120 protein boosts, formulated with either QS-21, Al(OH)_3_, MPL, or ISCOMATRIX™ adjuvant, at weeks 8 and 12. For an initial study examining the impact of two different DNA plasmid preparation methods, controls groups were immunized with only three pentavalent gp120 DNA immunizations, and received saline immunization in lieu of protein boosts.

### Differences in serum cytokines following immunization with gp120-expressing DNA plasmids in different preparations

In previous small animal studies of the DP6-001 DNA prime-protein boost formulation, gp120 DNA plasmid components were prepared using a regular plasmid kit as described above. Prior to the analysis of serum cytokines in response to the DP6-001 and candidate adjuvants, we aimed to rule out the potential contribution of residual endotoxin content in DNA plasmid preparations to the cytokine profiles observed. Mice received either three DNA immunizations followed by two saline boosts, or the full course of the DP6-001 DNA prime-protein boost vaccine regimen. DNA primes were prepared by either regular DNA plasmid kit or EndoFree DNA plasmid kit (referred to as EF DP6-001), as described above in Materials and Methods. Protein boosts were formulated with the QS-21 adjuvant, as with our previous studies of DP6-001 [5–7,27,30,31]. To characterize the systemic serum cytokines produced following adjuvanted protein immunization, in comparison to pre-immunized serum, we employed a multiplex cytokine array consisting of a panel of 12 cytokines including Th1 cytokines [Interleukin (IL)-2 and IFNγ], Th2 cytokines [IL-4], and pro-inflammatory cytokines [IL-1β, IL-6, RANTES (CCL5)]. In addition, we included cytokines and chemokines associated with activation and chemoattraction of monocytes, macrophages, NK cells, and granulocytes [MCP-1 (CCL2), MIP-1α (CCL3), MIP-1β (CCL4), G-CSF], neutrophils [KC (CXCL1)] and eosinophils [Eotaxin].

**Figure 2:**
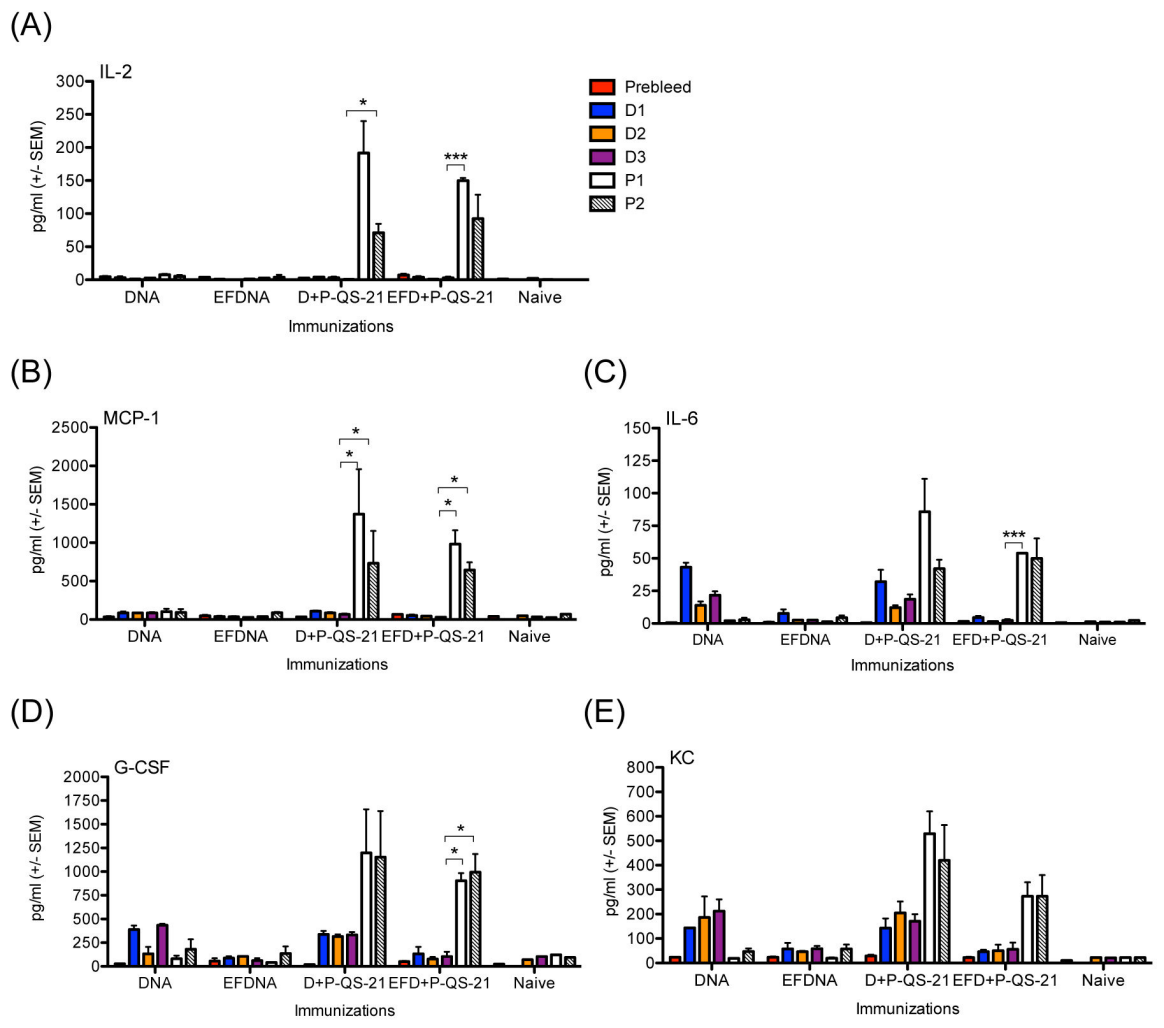
Temporal serum cytokine levels in mice immunized with endotoxin-free (EF) DNA prime compared to regular DNA plasmid preparation. Pentavalent gp120 DNA priming components were produced with either a regular or an EF DNA plasmid preparation kit. C57Bl/6 wild type mice were immunized with either three gp120 DNA primes and two “mock” saline boosts (‘DNA’ or ‘EF DNA’), or with three gp120 DNA primes and two gp120 protein boosts adjuvanted by QS-21 (‘D+P-QS-21’ or ‘EF D+P-QS-21’). Serum cytokines were quantified with a 12-plex Luminex panel in sera collected pre-immunization (‘Prebleed’), and at 6 hours after each of three DNA primes (‘D1,’ ‘D2,’ and ‘D3’) and two protein boost (‘P1,’ ‘P2’) immunizations. Shown are serum levels of (A) IL-2, (B) MCP-1, (C) IL-6, (D) G-CSF, and (E) KC. Statistical analysis was performed with a Student’s t test (*: p < 0.05, **: p < 0.01, ***: p < 0.001).

Overall, the different plasmid preparation methods employed did not substantially impact the levels of cytokine responses elicited at time points during the protein-adjuvant boost phase when the serum cytokine profiles will be measured. Eotaxin and MIP-1α were the only two cytokines moderately elevated in serum in mice immunized with regular DNA prep following the first protein boost as compared to EF DP6-001 but the difference dropped after the 2^nd^ protein boost (data not shown). Several of the analyzed cytokines detected in the serum clearly demonstrate the increased immune response associated with DNA prime-protein boost, as compared to immunization with DNA alone, seen in the comparison of serum cytokine levels 6 hours after the third DNA prime with the levels in serum 6 hours after each protein boost. Regardless of the method of DNA vaccine preparation, the serum levels of IL-2, IL-6, MCP-1, G-CSF, and KC were increased at 6 hours post-protein boost as compared time points 6 hours following the final DNA prime immunization (Figure 2).

During DNA priming immunizations, the serum levels of IL-2 (Figure 2A) and MCP-1 (Figure 2B) were low overall in mice primed with either DNA plasmid preparation. We observed a trend towards increased serum levels of IL-6 (Figure 2C), KC (Figure 2D), and G-CSF (Figure 2E) during DNA priming steps in mice immunized with regular DNA preparations as compared to EF preparations. However, these differences were not significant. Rather, serum levels of these cytokines were significantly boosted above DNA priming levels by protein boosts, while serum cytokine levels in mice that did not receive protein boosting dropped trended down towards background levels (Figure 2C–E). These results suggested that our future studies of adjuvanted protein-associated serum cytokine profiles in the context of DNA prime-protein boost should focus on time points 6 hours after protein-adjuvant boosting. Importantly, we did not observe any significant differences in these serum cytokines between regular and EF DNA preparations at time points 6 hours after protein boost. This indicates the minimal impact of potential endotoxin content associated with different DNA plasmid preparations on the serum cytokines we measured at our time points of interest.

Remaining cytokines were either not above the background levels during both after DNA and protein immunizations (IFNγ and IL-4), or were at low concentration only after DNA priming phase but not after protein immunization (MIP-1β and RANTES) (data not shown).

### Effect of DNA prep in gp120-encoding DNA plasmid on DP6-001 vaccine-induced Env-specific IgG response

**Figure 3:**
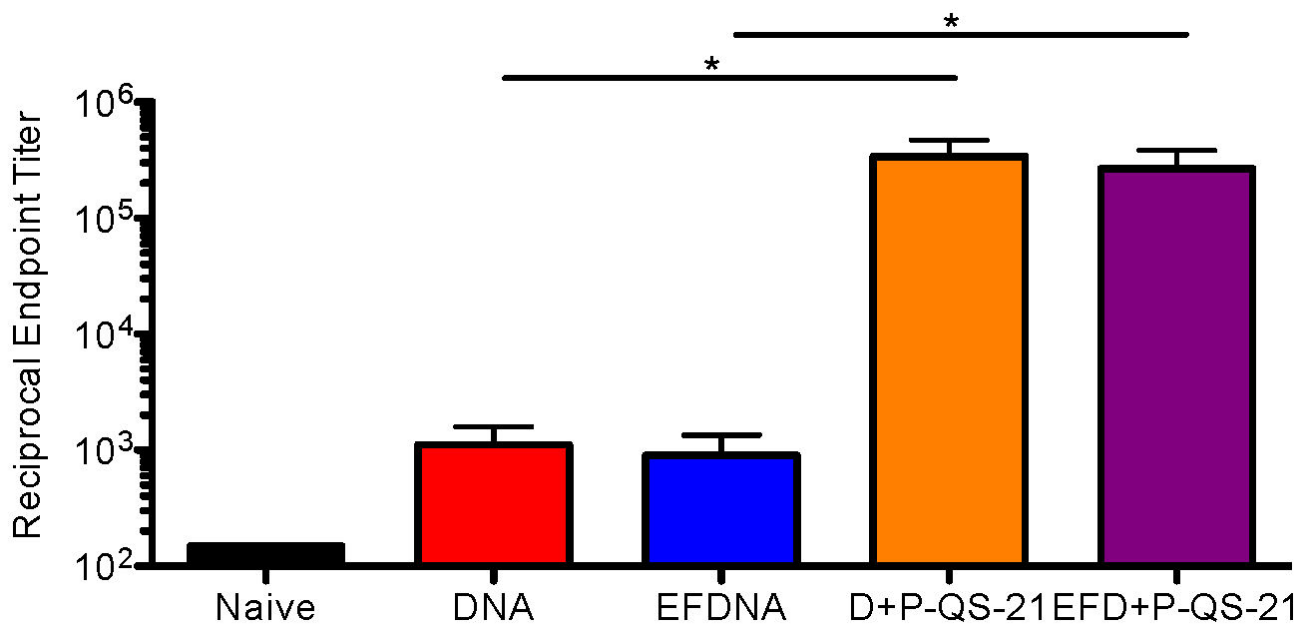
Endpoint gp120-specific IgG titer in C57Bl/6 mice immunized with endotoxin-free DNA prime versus regular DNA plasmid preparation. Total gp120-specific IgG was measured by ELISA in sera collected 7 days after the second protein boost, in week 13. Statistical analysis performed by one-way ANOVA and Tukey post-test (*: p<0.05).

In addition to examining nonspecific serum cytokine responses, we aimed to rule out the potential impact of gp120 DNA plasmid preparations on the Env-specific IgG antibody response observed in our adjuvant studies. Sera collected from the immunized mice 7 days following the final protein boost was used to determine the Env-specific IgG endpoint titer by ELISA (Figure 3). As expected, mice that received the full DP6-001 with adjuvanted protein boost demonstrated significantly higher IgG titers than mice that received DNA priming only, regardless of the method of g120 DNA plasmid preparation.

### Induction of Env-specific T cell responses by DP6-001 vaccine

**Figure 4:**
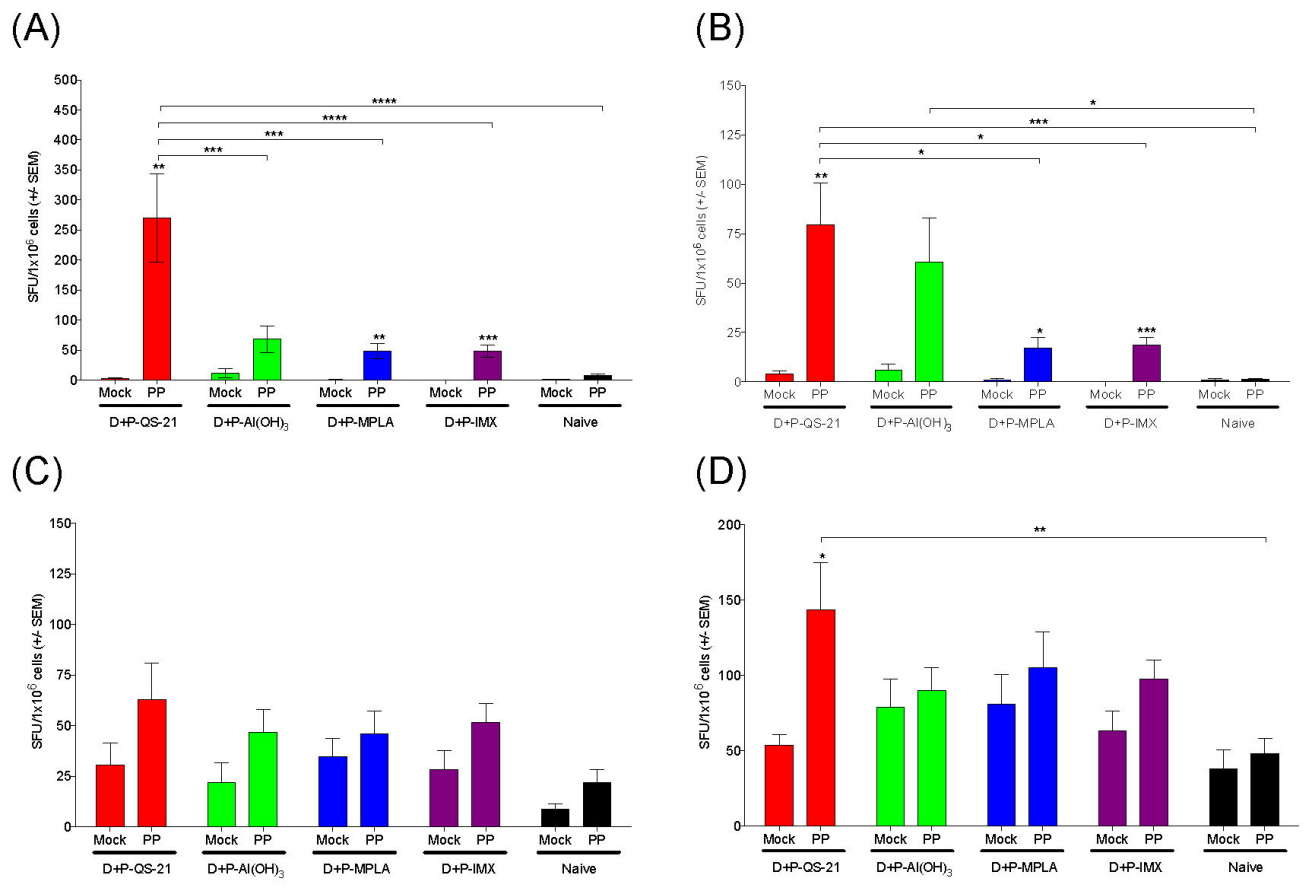
Env-specific cellular immune responses in splenocytes from mice immunized with DP6-001 and candidate adjuvants. Spleens were harvested from immunized C57Bl/6 wild type mice at termination 7 days after the final protein boost. Cells were cultured for 18 hours either receiving the stimulation of a truncated HIV-1 gp120 Clade B peptide pool (‘PP’) or media (‘mock’). Cytokine spot-forming units (SFU) per million splenocytes were visualized with a CTL Imager and analyzed with Immunospot™ software. Splenocyte production of gp120-specific Th1 cytokines (A) IFNγ and (B) IL-2, and gp120- specific Th2 cytokines (C) IL-4 and (D) IL-6 were measured. Statistical analysis of peptide stimulation over mock stimulation was calculated by Student’s t-test. Significant values (*: p < 0.05) are represented above error bars. Statistical differences in peptide stimulation between adjuvant groups were calculated by a Two-way ANOVA and Bonferroni post-test (*: p < 0.05, **: p < 0.01, ***: p < 0.001) (IMX = ISCOMATRIX™ adjuvant)..

Once we established the regular DNA prep was qualified for use in the current studies, T cell responses following immunization with DP6-001 with various candidate adjuvants were characterized in C57Bl/6 mouse splenocytes. ELISpot analysis was conducted to examine DP6-001 vaccine-induced production of Th1 and Th2 responses in the splenocytes of immunized mice, in response to a peptide pool (labeled ‘PP’) representing clade B Env consensus sequence [29]. Mice that received DP6-001 formulated with QS-21 most strongly produced an Env-specific IFNγ response, with much lower levels observed in mice immunized with protein formulated with Al(OH)_3_, MPLA, and ISCOMATRIX™ adjuvant (Figure 4A). All mice receiving adjuvanted protein formulations demonstrated a positive IL-2 response to peptides (Figure 4B) and minimal induction of IL-4 (Figure 4C) with no differences between groups. Baseline levels of Th2 cytokines IL-4 and IL-6 were notably elevated (Figure 4C, D). Mice receiving protein formulated with QS-21 demonstrated a positive but not significant induction of IL-6 over background, while those receiving protein formulated with all other adjuvants showed minimal induction of IL-6 (Figure 4D).

Intracellular cytokine staining (ICS) was also conducted to evaluate the functionality of vaccine-specific T cells by the production of Th1 and Th2 cytokines (IFNγ, IL-2 and IL-6) in response to a pool of overlapping peptides representing the clade B consensus Env sequence (Figure S1). No significant difference in CD4^+^ T cell cytokine induction was observed between adjuvanted protein groups. Similar to ELISpot results, mice immunized with protein formulated with QS-21 showed significant induction of Env-specific IFNγ (Figure S1A) and IL-6 (Figure S1C) by CD4^+^ T cells, and a marginally positive IL-2 (Figure S1B) response by CD4^+^ T cells. While there was a trend of positive Env-specific IFNγ responses by CD8^+^ T cells (Figure S1D), responses were not significantly induced above background, nor were there any significant differences between the different adjuvants. This result is not surprising as protein vaccines are not known for the induction of CD8^+^ T cell responses and DP6-001 vaccine was mainly designed for the induction of antibody responses.

### Eliciting Env-specific IgG antibody response using DP6-001 DNA prime and protein boost formulated with candidate adjuvants

In immunogenicity studies of DP6-001 in small animals and clinical volunteers, we reported the robust induction of vaccine-specific antibody response following immunization with the polyvalent Env DNA prime-protein boost formulation DP6-001 [5–7,27,30,31]. In our previous Phase I clinical study, DP6-001 protein boosts have been formulated with the saponin adjuvant, QS-21. In order to investigate the immunogenicity of alternative candidate adjuvants, particularly in comparison to QS-21, we immunized two strains of wild type mice with the pentavalent gp120 DNA prime-protein boost regimen employed in a phase I clinical trial [5–7]. Balb/c and C57Bl/6 mice were immunized with three DNA primes, followed by two protein boosts formulated with QS-21, Al(OH)_3_, MPLA, or ISCOMATRIX™ adjuvant according to the study design outlined in Figure 1. Sera were collected every two weeks after DNA immunization, and six hours post-protein boosts. Sera collected from mice seven days following the second protein boost were used to ascertain the endpoint titer of Env-specific IgG responses. For antibody responses, there is limited difference between Balb/c C57Bl/6 mice, and only data from C57Bl/6 wild type mice are presented.

**Figure 5:**
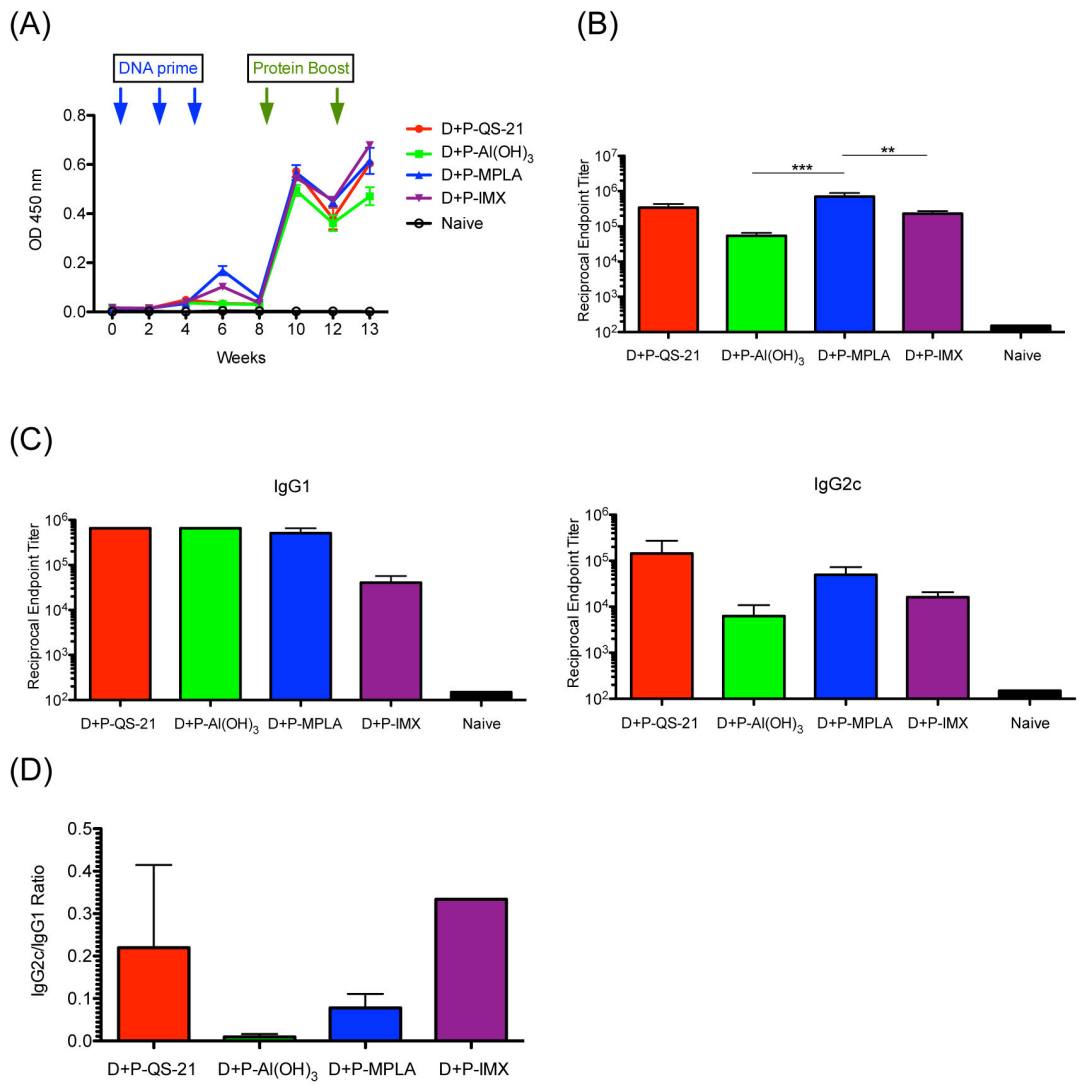
HIV-1 gp120-specific IgG response in C57Bl/6 wild type mice immunized with DP6-001 vaccine with different adjuvants. Total gp120-specific IgG was measured by ELISA in sera collected 7 days after final protein boost, in week 13. Protein boosts were formulated with QS-21 (red), Al(OH)_3_ (green), MPLA (blue), or ISCOMATRIX™ adjuvant (IMX) (purple). Naïve mice (black) received ‘mock’ saline injections in lieu of immunization. (A) Temporal gp120-specific IgG response was determined by ELISA using pooled sera samples from each group collected at two-week intervals. (B) gp120-specific endpoint IgG titer in was determined by ELISA using individual serum samples collected in week 13. (C) Endpoint IgG isotype profiles were determined by ELISA using individual serum samples collected in week 13. (D) Th1/Th2 ratio of IgG isotype responses was determined by comparing IgG2c/IgG1 ratio. Statistical significance was determined by one-way ANOVA and Tukey post-test (*: p < .05, **: p < .01, ***: p < .001).

Immunization with DP6-001 produced comparably robust gp120-specific IgG responses following the final protein boost, independent of the adjuvant used. Env-specific IgG levels were detectable following the third DNA immunization, and were significantly boosted by the first protein immunization. Levels of specific IgG dropped four weeks following the first protein boost, but were subsequently boosted by the second protein immunization (Figure 5A). Endpoint titer analysis showed that gp120-specific IgG levels were comparable in mice receiving formulations containing QS-21 or MPLA. IgG titers were significantly lower in mice immunized with formulations containing ISCOMATRIX™ adjuvant as compared to formulations containing MPLA and lower again in mice receiving formulations containing Al(OH)_3_ (Figure 5B).

In C57Bl/6 mice, sera collected 7 days after the final protein immunization was used to characterize the IgG isotype profiles of formulations with the candidate adjuvants. HIV-1 gp120-specific IgG1 titers were comparable between mice that received formulations containing QS-21, Al(OH)_3_, or MPLA adjuvants (Figure 5C). In comparison, IgG1 titers were slightly, but not significantly lower, in mice that received formulations containing ISCOMATRIX™ adjuvant. On the other hand, gp120-specific IgG2c titers were higher in mice immunized with formulations containing QS-21 and MPLA followed by formulations containing ISCOMATRIX™ adjuvant in comparison to mice that received formulations containing Al(OH)_3_ (Figure 5D). A ratio of the endpoint titers of Env-specific IgG2c, a correlate of Th1 responses, and IgG1, a correlate of Th2 responses, were used to demonstrate the Th1 vs. Th2 responses associated with each adjuvant (Figure 5D). Mice immunized with formulations containing Al(OH)_3_ demonstrated a low IgG2c/IgG1 ratio, and therefore predominantly Th2 response. In contrast, mice immunized with formulations containing MPLA or QS-21, and to a greater extent, ISCOMATRIX™ adjuvant, demonstrated a higher IgG2c/IgG1 ratio, indicating strong Th1 responses.

### Induction of unique serum cytokine profiles following protein boost with candidate adjuvants

Sera were collected throughout the DP6-001 immunization schedule in the current study, including time points prior to immunization at week 0, and also 6 hours following each protein-adjuvant boost at weeks 8 and 12. In order to characterize the serum cytokines produced following protein-adjuvant immunization, in comparison to pre-immunized serum, we employed a 12-plex cytokine array described above. By examining this panel of non-antigen specific, systemic cytokine responses in immunized mice 6 hours after each protein-adjuvant boost, our objective was to identify a unique profile of markers for each candidate adjuvant in the context of our prime-boost HIV vaccine. In addition, we will also identify cytokines and chemokines that are broadly induced by formulations containing the candidate adjuvants.

**Figure 6:**
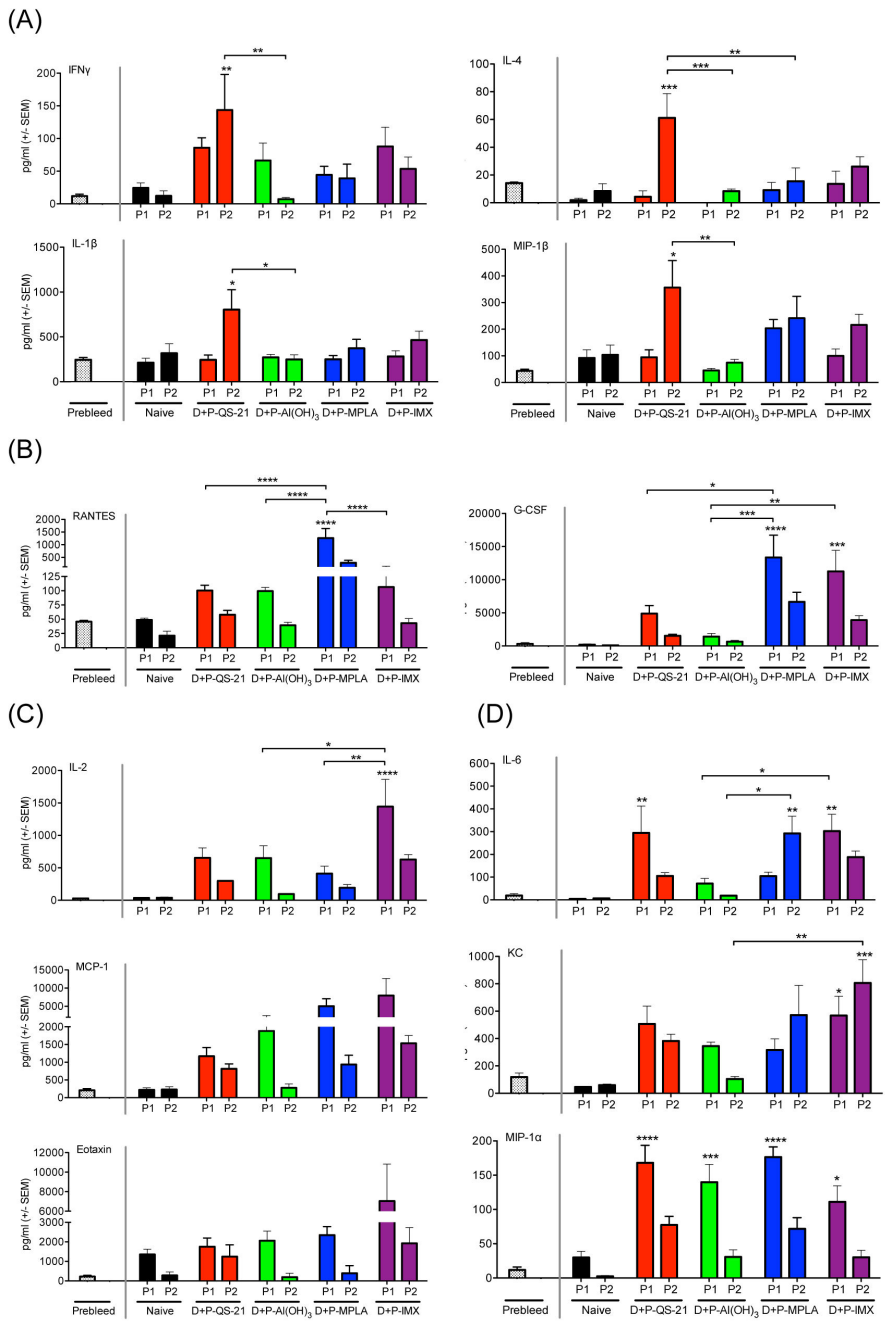
Serum cytokine concentration in mice vaccinated with DP6-001 and candidate adjuvants. C57Bl/6 wild type mice were immunized with DP6-001 DNA prime-protein boost (‘D+P’), with each protein boost formulated with QS-21 (D+P-QS-21), Al(OH)_3_ (D+P–Al(OH)_3_), MPLA (D+P-MPLA) or ISCOMATRIX™ adjuvant (D+P-IMX). Naïve mice received saline injections in lieu of immunization. Sera were collected pre-immunization and 6 hours following the first (P1) and second (P2) protein-adjuvant boosts. Cytokines were quantified in the serum of individual mice at a 1:4 dilution using a custom 12-plex Luminex panel. (A) QS-21 cytokine profile. (B) MPLA cytokine profile. (C) ISCOMATRIX™ adjuvant (IMX) cytokine profile. (D) Serum cytokines elevated following protein boost with formulations containing all candidate adjuvants. Significance over background is represented above error bars. Bracketed lines represent differences between adjuvant groups. Statistical significance was determined with a One-way ANOVA and Tukey post-test (*: p < .05, **: p < .01, ***: p < .001).

Animals immunized with DP6-001 including a boost of gp120 protein formulated with QS-21 demonstrated a unique serum cytokine profile, consisting of the Th1 cytokine IFNγ and the Th2 cytokine IL-4. Immunization with formulations containing QS21 was also associated with increased levels of the pro-inflammatory marker IL-1β, and the monocyte chemoattractant MIP-1β (Figure 6A). While levels of these four cytokines were relatively low in all adjuvant groups 6 hours after the first protein boost, they were clearly elevated in animals immunized with formulations containing QS21 following the second protein boost. Animals immunized with formulations containing QS-21 also demonstrated significantly higher IFNγ, IL-4, and IL-1β as compared to animals immunized with formulations containing Al(OH)_3_ or MPLA. Levels of MIP-1β, a chemoattractant for natural killer (NK) cells and monocytes, were significantly higher than in Al(OH)_3_-immunized animals. While mice immunized with formulations containing QS-21 showed significantly higher levels of IL-4 after the second protein boost in comparison to mice immunized with formulations containing ISCOMATRIX™ adjuvant, no significant differences were observed in other signature cytokines at this time point (Figure 6A).

Mice immunized with DP6-001 formulated with MPLA demonstrated significantly higher levels of the multifunctional and broadly acting chemokine RANTES in serum 6 hours after the first protein boost, as compared to other protein-adjuvant formulations (Figure 6B). Following the second protein-adjuvant boost, serum levels of RANTES were reduced in all groups, though in mice immunized with formulations containing MPLA the levels remained elevated compared to other formulations. In addition, levels of G-CSF were significantly increased in mice immunized with formulations containing MPLA following the first protein boost in comparison to mice immunized with formulations containing QS-21 or Al(OH)_3_ (Figure 6B).

Similarly, G-CSF was significantly increased at this time point in mice immunized with formulations containing ISCOMATRIX™ adjuvant in comparison to mice immunized with formulations containing QS-21 or Al(OH)_3_ (Figure 6B). G-CSF, which induces proliferation and differentiation of granulocytes, was characteristic of formulations containing either MPLA or ISCOMATRIX™ adjuvant in the context of the DP6-001 vaccine. In addition, animals receiving DP6-001 vaccine formulated with ISCOMATRIX™ adjuvant exhibited a unique profile of pro-inflammatory markers. The Th1 cytokine IL-2 was significantly increased following the first protein boost in mice immunized with formulations containing ISCOMATRIX™ adjuvant in comparison to formulations containing QS-21 or Al(OH)_3_. In addition, chemoattractants for eosinophils (Eotaxin), or monocytes and DCs (MCP-1), were also characteristic of formulations containing ISCOMATRIX™ adjuvant (Figure 6C). After the first protein boost, serum levels of MCP-1 in mice immunized with formulations containing QS-21 or Al(OH)_3_ were significantly lower compared to formulations containing ISCOMATRIX™ adjuvant, while levels in mice immunized with formulations containing MPLA were comparable. However, serum levels of Eotaxin were significantly increased in mice immunized with formulations containing ISCOMATRIX™ adjuvant in comparison to mice immunized with all other adjuvanted formulations.

Several serum cytokines and chemokines in our panel were shared among different candidate adjuvants in the context of HIV-1 gp120 DNA prime-protein boost vaccine. The neutrophil chemoattractant KC was strongly elevated in serum following protein boosts in mice immunized with DP6-001 formulated with QS-21, MPLA and ISCOMATRIX™ adjuvant, as compared to naïve and mice immunized with formulations containing Al(OH)_3_. There was no significant difference between these three adjuvanted formulations, although mice immunized with formulations containing ISCOMATRIX™ adjuvant demonstrated significantly higher levels of KC after the second protein boost as compared to formulations containing Al(OH)_3_ (Figure 6D).

IL-6, which may act as both a pro- and anti-inflammatory cytokine in response to a variety of stimuli, was strongly induced following protein boost in with formulations containing QS-21, MPLA, and ISCOMATRIX™ adjuvant, while levels in the mice immunized with formulations containing Al(OH)_3_ were very low. After the first protein boost, levels of IL-6 in mice immunized with formulations containing QS-21 and ISCOMATRIX™ adjuvant were both elevated comparably. Interestingly, in animals receiving protein boost formulated with MPLA, after one protein boost levels of IL-6 were significantly lower than those induced by formulations containing ISCOMATRIX™ adjuvant. By the second protein boost, however, levels of IL-6 induced by the formulations containing MPLA had risen to be comparable to initial levels seen with formulations containing ISCOMATRIX™ adjuvant, while the levels of IL-6 in mice immunized with formulations containing ISCOMATRIX™ adjuvant had notably fallen (Figure 6D).

Table 1 summarizes the serum cytokine profiles associated with each candidate adjuvant in the context of DP6-001, by comparing the fold-increase over background for each adjuvant.

**Table 1:** Trends in serum cytokine and chemokine induction over background following first and second protein-adjuvant boosts.

	D+P+QS-21		D+P+Al(OH)_3_		D+P+MPLA		D+P+IMX	
	P1	P2	P1	P2	P1	P2	P1	P2
IFNγ	+	++	+	-	+	+	+	+
IL-2	++	++	++	+	++	+	+++	++
IL-4	-	+	-	-	-	-	-	-
IL-6	++	+	+	-	+	++	++	+
IL-1β	-	+	-	-	-	-	-	-
Eotaxin	+	+	+	-	++	-	+++	+
KC	+	+	+	-	+	+	+	+
G-CSF	++	+	+	+	+++	++	+++	++
RANTES	+	-	+	-	++	+	+	-
MCP-1	+	+	+	-	++	++	+++	+++
MIP-1α	++	+	++	+	++	+	+	+
MIP-1β	+	+	-	-	+	+	+	+

Profiles Were Determined by Calculating the Fold Increase of Individual C57BL/6 Wild Type Mice in Each Group over Their Respective Pre-Immunization Cytokine Levels. Data Is Representative of Cytokine Data Shown In Figure 6. <2-Fold Increase: -. 2-10-Fold Increase: +. 10-30-Fold Increase: ++. >30-Fold Increase: +++. (IMX = ISCOMATRIX™ Adjuvant)

### Induction of serum cytokines by a protein-adjuvant vaccine in the absence of DNA prime

In order to define the effect of DNA priming on the protein and adjuvant serum cytokine profiles, additional study was performed in C57Bl/6 wild type mice that received only two DP6-001 protein boosts formulated with candidate adjuvants without DNA prime. Mice were immunized at weeks 0 and 4, and sera were collected for cytokine analysis at 6 hours after each protein boost. Serum cytokines were quantified in immunized mice in comparison to naïve mice using the 12-plex array described above.

**Figure 7:**
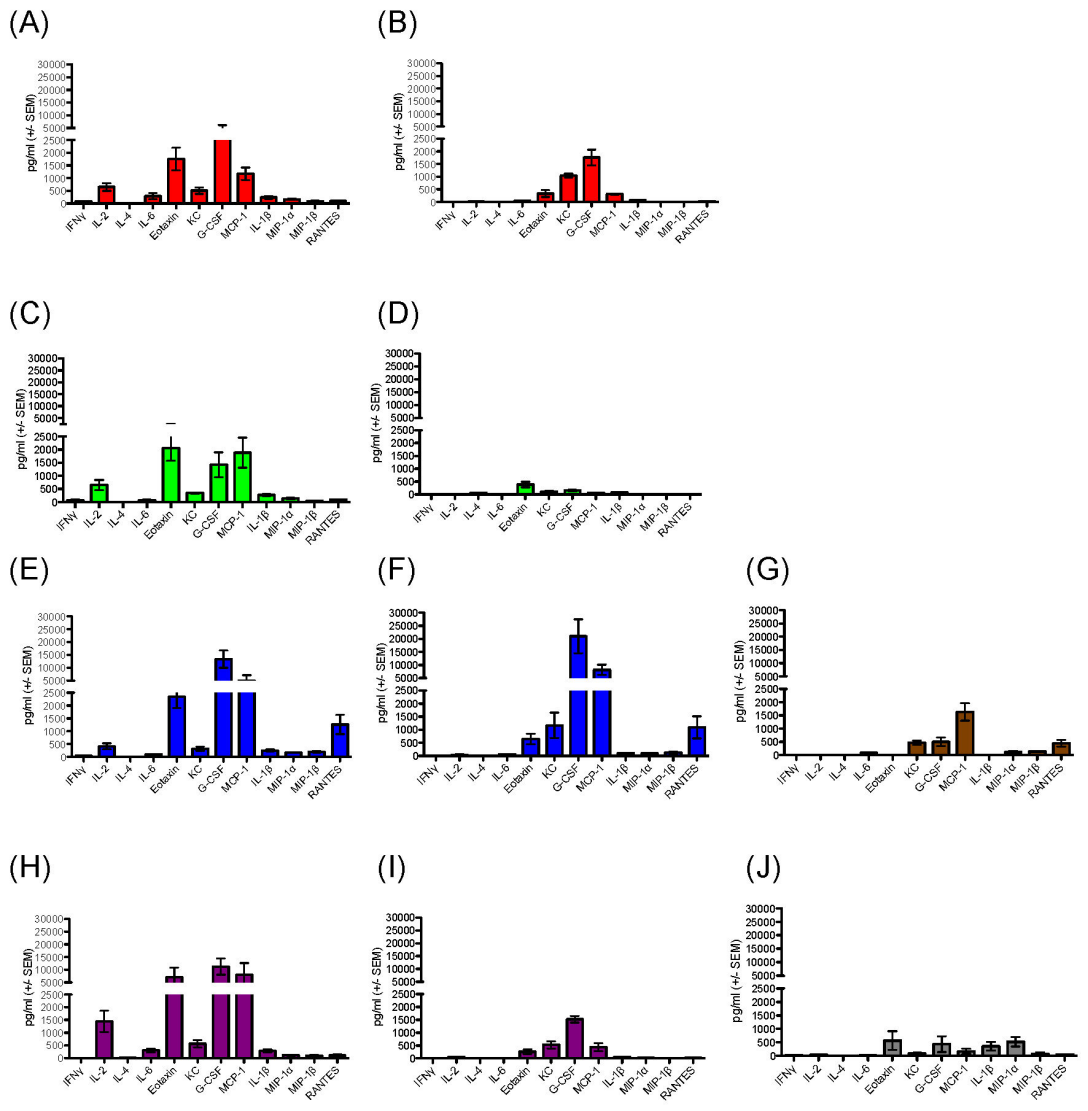
Compiled serum cytokine panels following the first protein boost in mice vaccinated with Env protein formulated with QS21 or Al(OH)_3_. C57Bl/6 wild type mice vaccinated with protein-only vaccine (‘P’) received immunizations at weeks 0 and 4 with the DP6-001 protein. Cytokines were quantified in serum from individual mice collected 6 hours following protein boost by a 12-plex Luminex array. Shown are serum cytokine levels 6 hours after the first protein-adjuvant boost. Mice were immunized with either DP6-001 prime-boost vaccine and adjuvants, a protein-only vaccine with adjuvants, or a vector-primed protein boost with adjuvants. (A) D+P-QS-21. (B) 2P-QS-21. (C) DP6-001-Al(OH)_3_. (D) 2P–Al(OH)_3_. (E) DP6-001-MPLA. (F) 2P-MPLA. (G) V+P-MPLA. (H) D+P-IMX. (I) 2P-IMX. (J) V+P-IMX.

Generally, serum cytokine levels associated with a protein-only vaccine were reduced in comparison to those observed in mice immunized with the complete DP6-001 DNA prime-protein boost regimen. Protein-only vaccine formulated with QS-21 results in low to background levels of Th1 and Th2 cytokines, including the previously observed QS-21-associated signature cytokines, IFNγ and IL-4 (Figure 7A-B). The inflammatory cytokine IL-1β and the chemokine MIP-1β, associated with QS-21 in the context of DP6-001 prime-boost vaccine, were substantially reduced after immunization with a protein-only vaccine (Figure 7A-B). Similarly, protein vaccine formulated with QS-21 demonstrated moderate reductions in the chemokines G-CSF, MCP-1, MIP-1α, and RANTES (Figure 7A-B). While DP6-001 prime-boost vaccine formulated with Al(OH)_3_ produced overall low, unimpressive cytokine profiles (Figure 7C), we observed that protein-only immunization with formulations containing Al(OH)_3_ also demonstrated even lower or negligible levels of Th1/Th2 cytokines, inflammatory cytokines, and chemokines (Figure 7D).

Similar studies with protein alone vaccination were conducted with formulations containing MPLA and ISCOMATRIX™ adjuvant, but also included groups that received three empty DNA vector as the prime followed with protein boost (Figure 7). In the context of a prime-boost vaccine, formulations containing MPLA were primarily characterized by potent induction of G-CSF and RANTES (Figure 7E). Interestingly, while Th1/Th2 cytokines and other chemokines are notably reduced following a protein-only vaccine formulated with MPLA, the MPLA formulation-associated signature chemokines G-CSF and RANTES, as well as MCP-1 and KC, are minimally changed in the absence of DNA priming (Figure 7F). Vector-primed mice immunized with protein-MPLA demonstrated reduced cytokines and chemokines (Figure 7G) in comparison to mice immunized with the complete DP6-001 regimen (Figure 7E).

Of the signature cytokines associated with formulations containing ISCOMATRIX™ adjuvant in the context of the DP6-001 prime-boost vaccine, such as IL-2, Eotaxin, G-CSF and MCP-1 (Figure 7H), IL-2, Eotaxin, and MCP-1 were substantially reduced in mice immunized with protein-only vaccine containing ISCOMATRIX™ adjuvant (Figure 7I). Though still above pre-immunization levels, serum levels of G-CSF were reduced in the absence of a DNA prime (Figure 7I). Control mice immunized with three empty vector DNA primes followed by DP6-001 protein boosts formulated with ISCOMATRIX™ adjuvant, however, demonstrated low or negative levels of all of the serum cytokine and chemokines analyzed (Figure 7J).

In summary, from our analysis of serum cytokines associated with candidate adjuvants formulated with the DP6-001 prime-boost vaccine, several markers, IL-6, KC, and MIP-1β, were identified that were comparably induced following protein boosting with formulations containing either QS-21, MPLA, or ISCOMATRIX™ adjuvant. Consistent with the trend of reduced cytokines associated with the absence of DNA priming, both IL-6 and MIP-1β were reduced or negative in comparison to DNA primed animals. A notable exception to this trend was the neutrophilic chemoattractant KC, which remained at comparable levels regardless of vaccine regimen (Figure 7). The inclusion of protein-only vaccines and empty vector DNA prime controls allows us to confirm that the cytokine and chemokine profiles associated with our candidate adjuvants are unique to the context of a DNA prime-protein boost vaccine strategy, and confirmed the concept that the improved immunogenicity based on serum cytokines was due to the inclusion of DNA priming steps.

## Discussion

In recent years, the heterologous prime-boost vaccination strategy has demonstrated considerable advantages over the classical vaccine strategies based on homologous prime-boost strategies. Results from RV144 clinical trial of viral vector prime and recombinant gp120 protein boost demonstrated unprecedented partial protection against the transmission of HIV-1 [1–4]. Similarly, we have previously reported that the DP6-001 vaccine formulation, consisting of a DNA prime and protein boost, was highly immunogenic in both preclinical and clinical studies [5–7,32]. A Phase I clinical trial of DP6-001 vaccine showed the generation of balanced Env-specific T cell and high titer Env-specific antibody responses including broadly neutralizing antibodies in study vaccinees [7].

In both of the above studies, the Env protein boost is now recognized as a critical component. However, the roles of adjuvant in such a protein boost immunization remains unclear. Protein vaccines are formulated with adjuvants with the goal of promoting and harnessing the innate non-antigen specific, pro-inflammatory response, and ultimately enhancing the antigen-specific adaptive immune response. This strategy is not without the risk of adverse effects. Out of candidate adjuvants in the current study, only Al(OH)_3_ and MPLA are components in licensed vaccines in the United States. Even well established and tolerable vaccine adjuvants such as Al(OH)_3_ must be thoroughly evaluated for both efficacy and safety in the context of new vaccine formulations before being approved for clinical use. Vaccine antigen, dosing, route of administration, and schedule may all impact the immunogenic profile as well as the reactogenicity of a given adjuvant. In addition, adjuvants must be selected with the pathogen-specific requirements of protection in mind. While sterilizing immunity against HIV-1 infection requires a strong humoral response including neutralizing antibodies, lessons from preclinical studies have shown that CD8^+^ and CD4^+^ T cell responses are important in facilitating both the humoral and cell-mediated immunity induced by an HIV-1 vaccine.

In the current study, three well-studied adjuvants, QS-21, MPLA, and ISCOMATRIX™ adjuvant, in addition to the widely used Al(OH)_3_ adjuvant as a control, all demonstrated comparable immunogenicity in the context of the DP6-001 DNA prime-protein boost in a mouse model. With the exception of Al(OH)_3_, the other three potent adjuvants each strongly induced a vaccine-specific antibody response. While the QS-21 adjuvant was associated with the strongest induction of Env-specific CD4^+^ T cell responses by intracellular cytokine staining and ELISpot assay, there were no significant differences in Env-specific Th1 and Th2 responses between the adjuvants evaluated as determined by ICS. Env-specific T cell ELISpot results showed that formulations containing QS-21 induced significantly greater Th1 responses as compared to other adjuvants. Formulations containing MPLA and ISCOMATRIX™ adjuvant induced positive specific Th1 responses to a lesser extent than QS-21. In contrast, all adjuvant formulations could elicit comparably weak Env-specific Th2 responses.

While antigen specific responses were similar for formulations containing each of the adjuvants included in the current study, the serum cytokine profiles were quite different. Multiplex arrays for nonspecific serum cytokines proved an invaluable tool in detecting differences in the non-Env specific innate responses to the DP6-001 vaccine and candidate adjuvants. In the context of the DP6-001 prime-boost vaccine, the candidate adjuvants were easily distinguished by these cytokine profiles and chemoattractants indicative of differential immune cell recruitment. The local and systemic inflammatory environment induced by an adjuvant formulated with vaccine requires a delicate balancing act. Early resident and recruited innate immune cells release of a complex milieu of cytokines and chemoattractants that together influence subsequent waves of immune cell infiltrates, and thus enhance antigen uptake and presentation [33]. Ultimately, this microenvironment shapes the antigen-specific adaptive immune response, directing a Th1 or Th2 CD4^+^ T cell bias and enhancing B cell function and antibody production. However, overstimulation of a pro-inflammatory response may result in undesirable local and systemic symptoms, including hypersensitivity reactions, fever and myalgia.

The multifunctional cytokine IL-6 is a central player in inflammation. Along with IL-1β, IL-6 promotes the acute phase response and fever [34]. IL-6 also mediates neutrophilic inflammation and the shift from innate acute inflammation to chronic inflammation and adaptive immunity [35–38]. In the current study, 6 hours following protein boost, we observed that IL-6 was universally induced to comparable levels by formulations containing each candidate adjuvant. This pattern was also observed for the neutrophil chemoattractant, KC. These serum cytokine levels associated with formulations containing the control adjuvant, Al(OH)_3_, were typically low compared to all other groups. MIP-1α, which, like MIP-1β, is produced by macrophages to promote neutrophilic inflammation and immune cell recruitment [39], was produced comparably by all adjuvanted formulations including those with Al(OH)_3_, but at levels not significantly elevated above background. These cytokines characteristic of DP6-001 DNA prime-protein boost with all adjuvants reflects an early, acute inflammatory response to immunization, likely predominated by neutrophils.

In the current study, formulations containing QS-21 were associated with the Th1 cytokine IFNγ and the Th2 cytokine IL-4, supporting our findings of mixed Env-specific Th1/Th2 T cell responses and IgG isotyping, as well as previous reports of *Quillaia* saponin fractions [40]. Elevated serum MIP-1β was characteristic of only formulations containing QS-21. IL-1β, which plays a major role in the acute phase response and fever [34], as well as neutrophilic inflammation [39,41], was also strongly associated with formulations containing QS-21. It may be a point of interest that these characteristic cytokines were most strongly observed following the second protein boost, rather than following the first protein boost, as was the pattern for other adjuvants. This may be suggestive of a progressive inflammatory response with subsequent immunizations.

By comparison, the more clinically tolerable saponin formulation ISCOMATRIX™ adjuvant was characterized by strong IL-2, which is supportive of an expected Th1 and CD8^+^ T cell response. Elevated systemic IL-6 as well as Eotaxin, a chemoattractant for eosinophils, are indicative of a Th2 response, supporting the reported mixed Th1/Th2 profile of ISCOMATRIX™ vaccines [12,13,15,16,42,43]. Granulocyte factor G-CSF and MCP-1, which recruits monocytes and macrophages subsequent to early neutrophil infiltration [44], were also associated with the protein boost containing ISCOMATRIX™ adjuvant. The distinct cytokine and chemokine pattern observed here with DP6-001/ISCOMATRIX™ adjuvant consists of Th1 and Th2 cytokines as well as chemoattractants indicative of the recruitment of monocytes, macrophages, NK cells, and granulocytes. This profile supports a serum cytokine profile recently reported by Wilson et al. 6 hours following subcutaneous administration of ISCOMATRIX™ adjuvant without antigen [45]. Our profile of DP6-001/ISCOMATRIX™ adjuvant is consistent with a profile of serum cytokines and immune cell infiltration at draining lymph nodes described by Duewell et al., consisting of B and T cells, DCs, NK cells, and granulocytes, in mice subcutaneously immunized with OVA antigen and ISCOMATRIX™ adjuvant [46].

DP6-001 formulated with MPLA demonstrated high serum levels of granulocyte factor G-CSF, in addition to elevated serum RANTES, a T cell-produced chemoattractant for eosinophils, T cells, and NK cells. The serum cytokine profile of MPLA observed in the context of the DP6-001 Env DNA prime-protein boost may support previous evaluations of MPLA formulations in a mouse model. Mata-Haro et al. previously reported that in the hours following injection, MPLA formulated with OVA antigen was correlated with serum G-CSF and MCP-1, and low but positive RANTES, as compared to LPS. MyD88-associated IL-6 and MIP-1α were weakly positive, while IFNγ and IL-1β were minimal [22].

We have described here a complex picture of the differential immune responses elicited by each candidate adjuvant in the context of a novel heterologous prime-boost vaccine, with the goal of identifying correlates of immunogenicity and markers of reactogenicity that may aid in the selection of an adjuvant for future optimized vaccine formulations. While the immunogenicity of the formulation containing our previously employed adjuvant QS-21 was comparable to formulations with our other candidate adjuvants, we may be able to correlate a unique systemic inflammatory response associated with QS-21. Formulations with all three potent adjuvants, with the exception of Al(OH)_3_, demonstrated comparable serum levels of IL-6 and KC, and low but positive levels of MIP-1α. Beyond this, the additional pro-inflammatory environment of serum IL-1β and MIP-1β associated specifically with QS-21 may contribute to reported adverse events. The predominance of markers for acute inflammation and fever, as well as products and mediators of neutrophilic inflammation, is of particular interest given the nature of a previously reported vasculitis associated with DP6-001 and QS-21 [6]. Leukocytoclastic vasculitis is due to the toxic effect of neutrophilic degranulation products on the endothelial cells of small vasculature [47].

In contrast to QS-21, formulations containing MPLA and ISCOMATRIX™ adjuvant likely demonstrate potentially milder reactogenic profiles. Both MPLA and ISCOMATRIX™ adjuvant are largely characterized by broadly acting chemoattractants recruiting a varied population of immune cells, including granulocytes, NK cells, monocytes, DCs, and macrophages. However, formulations containing either of these adjuvants demonstrate notably lower levels of additional inflammatory cytokines and chemokines such as IL-1β and MIP-1β in comparison to QS-21.

In the process of evaluating different adjuvants with the DP6-001 formulation, we observed that serum cytokine responses demonstrate unique kinetics in a prime-boost vaccine regimen. The optimal time to detect high level cytokine responses was at 6 hours after the protein boost, while early studies demonstrated low to negative responses at the end of DNA priming immunization. Repeated boost with protein-adjuvant vaccines actually led to a reduced cytokine response following subsequent protein boosts, except with the use of QS-21.

In the current study, we performed several control immunizations to confirm the correlation of our unique adjuvant serum cytokine profiles to their use in the context of a unique DNA prime-protein boost vaccine strategy. In animals immunized with a protein-only vaccine formulated with either QS-21, Al(OH)_3_, MPLA, or ISCOMATRIX™ adjuvant, serum cytokine and chemokine panels were largely reduced in comparison to those in DNA-primed animals. A similar trend was observed in animals that received an empty vector DNA prime and protein boost formulated with our candidate adjuvants. These serum cytokine profiles from protein-only or empty vector primed vaccine formulations suggest that the serum cytokine and chemokine profiles we have defined for QS-21, MPLA, and ISCOMATRIX™ adjuvant are unique to the context of a DNA prime-protein boost vaccine strategy. Furthermore, the poor serum cytokine response induced by empty vector DNA priming in comparison to DP6-001 DNA priming suggests that the antigen itself encoded in the DNA prime plays an important role in the response induced by subsequent boosting with protein antigen. Certainly DNA plasmids themselves may activate innate nucleic acid sensing pathways in a non-antigen specific manner and influence the ultimate adaptive immune response in a manner that is yet to be understood. The immune response to priming with DNA plasmids encoding antigen results in the generation of an antigen-specific adaptive immune response, which shapes the nature of the immune response to subsequent boosts, in concert with the action of adjuvants on innate immune pathways. That we observe this difference at such an early time point after boosting immunization may be suggestive of a memory response. Indeed, this data prompts further questions about how DNA vaccines act on innate and adaptive immunity, as well as how both the encoded antigen and the DNA itself act to shape a setting for subsequent heterologous boosts.

A few notable exceptions to this trend were observed. In mice immunized with protein formulated with MPLA, the markers G-CSF and RANTES, associated with MPLA in the context of the full DP6-001 regimen, were apparently uncompromised in the absence of DNA priming. In addition, the neutrophil chemoattractant KC remained at relatively unchanged levels in the context of formulations containing QS-21, MPLA, or ISCOMATRIX™ adjuvant, regardless of vaccination strategy. This observation suggests that an early immune response to vaccination characterized by strong neutrophilia was common to all tested vaccine strategies and adjuvants, and was minimally impacted by the presence or absence of DNA priming.

The current study also suggested that while trace amount of endotoxin contaminants will lead to elevated levels of only a few cytokines, this has a minimal effect on the overall levels of Env-specific antibody responses. These findings ruled out the potential contribution of endotoxin contamination to vaccine-induced immunogenicity and the observed profiles of non-antigen specific serum cytokines. This analysis confirmed that the adjuvant effects we observed with our various DP6-001 formulations were related to the adjuvant used, and not potentiated by method of DNA preparation.

In summary, we have reported that two potent candidate adjuvants MPLA and ISCOMATRIX™ adjuvant demonstrate comparable immunogenicity to our previously employed adjuvant QS-21, in the context of a heterologous, multiclade HIV-1 Env DNA prime-protein boost regimen. However, these adjuvants differ considerably in terms of induction of pro-inflammatory cytokines and chemokines responsible for local and systemic immune cell recruitment. This study provides critical insight about these adjuvants in formulation with Env antigen, as well as in the context of a DNA-primed immune system. In addition, the distinct cytokine and chemokine profiles defined for each adjuvant shed light on useful correlates of vaccine immunogenicity as well as pro-inflammatory markers of reactogenicity for guidance in adjuvant selection for optimized Env prime-boost formulations.

## Supporting Information

Figure S1 **Magnitude of Env-specific CD4^+^ and CD8^+^ T cell responses induced by DP6-001 immunization and adjuvants in C57Bl/6 wild type mice.** Cytokines were analyzed in murine splenocytes 7 days after final protein boost. Spleens were harvested at termination 7 days after the final protein boost. Splenocytes were cultured for 5 hours either receiving the stimulation of a consensus HIV-1 gp120 Clade B peptide pool (‘Env-B’) or media (‘baseline’). Env-specific cytokine production by T cells was quantified by intracellular cytokine staining and samples were run on an LSR II FACS machine. Data was analyzed using FlowJo software. Shown is the production of (A) IFNγ, (B) IL-2, and (C) IL-6 by CD4^+^ T cells, and (D) IFNγ by CD8^+^ T cells in mice vaccinated with DP6-001 and candidate adjuvants. Statistical comparisons between adjuvant groups were performed with a one-way ANOVA and Tukey post-test. Statistical significance of antigen-specific responses over background was performed with a Student’s t-test.
(TIF)
