# Supplementary figures and images for: Serum Cytokine Profiles Associated with Specific Adjuvants Used in a DNA Prime-Protein Boost Vaccination Strategy
Source: PLoS One. 2013 Sep 3;8(9):e74820. doi: 10.1371/journal.pone.0074820 (PMC3760864; doi:10.1371/journal.pone.0074820)

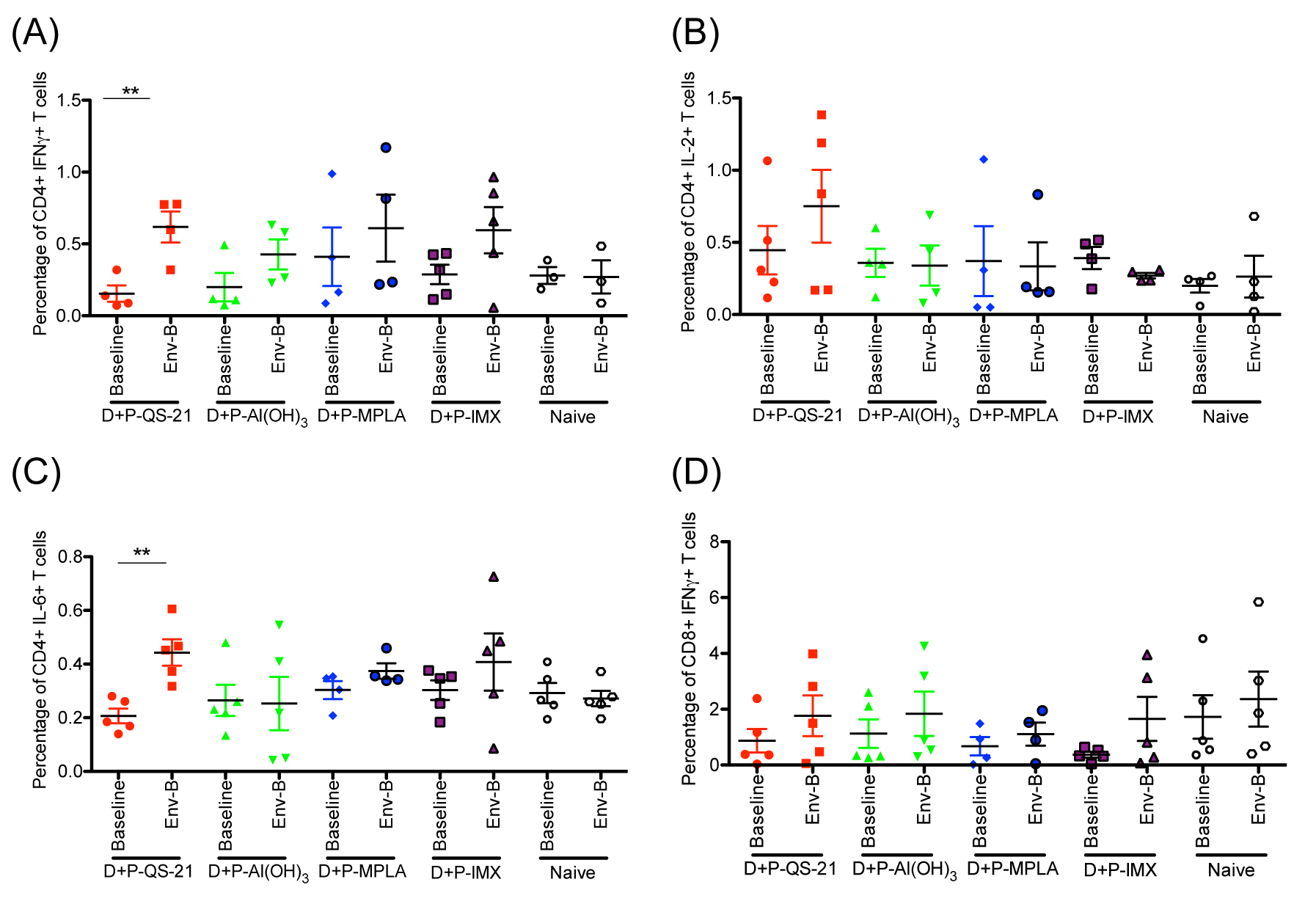

Supplement: Figure S1 — Magnitude of Env-specific CD4+ and CD8+ T cell responses induced by DP6-001 immunization and adjuvants in C57Bl/6 wild type mice. Cytokines were analyzed in murine splenocytes 7 days after final protein boost. Spleens were harvested at termination 7 days after the final protein boost. Splenocytes were cultured for 5 hours either receiving the stimulation of a consensus HIV-1 gp120 Clade B peptide pool (‘Env-B’) or media (‘baseline’). Env-specific cytokine production by T cells was quantified by intracellular cytokine staining and samples were run on an LSR II FACS machine. Data was analyzed using FlowJo software. Shown is the production of (A) IFNγ, (B) IL-2, and (C) IL-6 by CD4+ T cells, and (D) IFNγ by CD8+ T cells in mice vaccinated with DP6-001 and candidate adjuvants. Statistical comparisons between adjuvant groups were performed with a one-way ANOVA and Tukey post-test. Statistical significance of antigen-specific responses over background was performed with a Student’s t-test. (TIF) [file pone.0074820.s001.tif]
